# An Adaptive Biosystems Engineering Approach towards Modeling the Soluble-to-Insoluble Phase Transition of Clofazimine

**DOI:** 10.3390/pharmaceutics14010017

**Published:** 2021-12-22

**Authors:** Andrew R. Willmer, Steven Dunne, Rosemary Swanson, Deepak Almeida, Nicole C. Ammerman, Kathleen A. Stringer, Edmund V. Capparelli, Gus R. Rosania

**Affiliations:** 1 Department of Pharmaceutical Sciences, College of Pharmacy, University of Michigan, Ann Arbor, MI 48109, USA; awillmer@umich.edu; 2 Department of Chemistry, University of Michigan, Ann Arbor, MI 48109, USA; scdunne@umich.edu; 3 Johns Hopkins Center for Tuberculosis Research, Johns Hopkins University School of Medicine, Baltimore, MD 21205, USA; swanson.rose@gmail.com (R.S.); dalmeid3@jhmi.edu (D.A.); nicole.ammerman@jhu.edu (N.C.A.); 4 Department of Medical Microbiology and Infectious Diseases, Erasmus MC, University Medical Center Rotterdam, 3015 GD Rotterdam, The Netherlands; 5 Department of Clinical Pharmacy, College of Pharmacy, University of Michigan, Ann Arbor, MI 48109, USA; stringek@med.umich.edu; 6 Department of Pediatrics and Skaggs School of Pharmacy and Pharmaceutical Science, University of California, San Diego, CA 92093, USA; ecapparelli@ucsd.edu

**Keywords:** modeling and simulation, pharmacokinetics, small molecules, drug targeting, drug delivery

## Abstract

Clofazimine (CFZ) is a weakly basic, small-molecule antibiotic used for the treatment of mycobacterial infections including leprosy and multidrug-resistant tuberculosis. Upon prolonged oral administration, CFZ precipitates and accumulates within macrophages throughout the host. To model the pharmacokinetics of CFZ, the volume of distribution (Vd) was considered as a varying parameter that increases with continuous drug loading. Fitting the time-dependent change in drug mass and concentration data obtained from CFZ-treated mice, we performed a quantitative analysis of the systemic disposition of the drug over a 20-week treatment period. The pharmacokinetics data were fitted using various classical compartmental models sampling serum and spleen concentration data into separate matrices. The models were constructed in NONMEM together with linear and nonlinear sigmoidal expansion functions to the spleen compartment to capture the phase transition in Vd. The different modeling approaches were compared by Akaike information criteria, observed and predicted concentration correlations, and graphically. Using the composite analysis of the modeling predictions, adaptive fractional CFZ sequestration, Vd and half-life were evaluated. When compared to standard compartmental models, an adaptive Vd model yielded a more accurate data fit of the drug concentrations in both the serum and spleen. Including a nonlinear sigmoidal equation into compartmental models captures the phase transition of drugs such as CFZ, greatly improving the prediction of population pharmacokinetics and yielding further insight into the mechanisms of drug disposition.

## 1. Introduction

Clofazimine (CFZ) is an antimycobacterial agent used to treat leprosy and multidrug-resistant tuberculosis alongside some non-tuberculosis mycobacterial infections. Recently, CFZ has also been found to inhibit SARS-CoV-2 infection in vitro and in animal models [1,2], and its efficacy is currently being tested in a phase II clinical trial [3]. The precipitation and accumulation of this weakly basic drug in the lysosomes of macrophages is a phenomenon that has raised considerable interest in the drug targeting and delivery field, since it corresponds to the most potent, cell-specific drug targeting mechanism that has been discovered to date [4,5]. The accumulation of CFZ in macrophage lysosomes has been shown to occur by thermodynamically favorable conditions that lead to the precipitation of drugs by pH-dependent ion trapping. The protonated species of the weak base in the lysosome of the macrophage, together with the formation of the very insoluble hydrochloride (CFZ-HCl) salt, form a highly stable crystal upon interaction of the protonated drug with chloride [6,7,8]. In macrophage lysosomes, the hydrochloride crystals are found within membrane-associated complexes referred to as crystal-like drug inclusions (CLDIs). This phenomenon has been observed throughout the body, but it occurs primarily in macrophages of the spleen, liver, gut, and lungs. The large amount of CFZ that accumulates in macrophages over time directly leads to a pronounced expansion of the volume of distribution (Vd) of the drug [9].

Although it has been used clinically for many decades [10,11], CFZ pharmacokinetics are characterized by an increasing, dose-dependent half-life and high interindividual variability [12], which complicates pharmacokinetics analysis. In an experimental mouse model used to analyze the mechanisms underlying the complex pharmacokinetics of CFZ, the dynamic half-life of CFZ was associated with the formation and accumulation of hydrochloride salt as CLDIs grew and expanded within macrophage lysosomes [6,13]. This increasing accumulation was accompanied by changes in the distribution of the drug within the organism, as well as the formation of local drug depots that remained long after treatment stopped [6]. While the soluble phase of CFZ is the pharmacologically active moiety that can freely exert antibacterial or antiviral activity [1,2,10], the CLDI drug depot could be beneficial since it concentrates in macrophages, which are directly involved in combating infection. Crystal-like drug inclusions have also been shown to increase with repeated dosing in both mice and humans [6]. Due to the high concentration of macrophages in the spleen, this organ sequesters CFZ at 10 to 100 times the concentration of other tissues [6,14].

Recently, the pharmacokinetics of clofazimine in tuberculosis patients was described as a three-compartment model [12]. In order to improve upon the standard compartmental modeling approaches used to analyze the pharmacokinetics of CFZ, we sought to develop an adaptive pharmacokinetics modeling approach that would better capture the soluble-to-insoluble phase transition and subsequent increasing Vd of the drug, and to compare its predictive accuracy to a standard compartmental model. Using mice as a model organism, our analysis of CFZ pharmacokinetics during a 20-week treatment period demonstrates how such an adaptive pharmacokinetics modeling approach can provide insights into the changes in serum and spleen concentrations measured during prolonged treatment, increasing our understanding of the pharmacokinetics parameters that explain the context-dependent accumulation of CFZ.

The results reported herein are the first time that volume of distribution changes resulting from soluble-to-insoluble phase transitions of drug molecules are directly modeled and analyzed, using a population pharmacokinetics modeling approach that should be applicable to the study of CFZ pharmacokinetics in the human population. The significance of this analysis is not only relevant to improving our understanding of the mechanistic underpinning of CFZ pharmacokinetics, but it will help advance the development of macrophage-targeted drugs and the design of self-organizing drug depot systems.

## 2. Materials and Methods

### 2.1. Data Acquisition and Compartmental Pharmacokinetic Modeling

Individual mouse data were obtained from a previously published study in which CFZ concentrations were measured in the serum and spleen of healthy BALB/c mice given 25 mg/kg of CFZ by oral gavage once daily, Monday through Friday, over a 20-week period, together with measurements of the mass of the organ. Three mice were sacrificed then sampled at seven discrete intervals (7, 14, 28, 56, 84, 114 and 140 days) after starting therapy and prior to administering the daily dose. CFZ concentrations were measured by liquid chromatography/mass spectrometry (LC/MS) using a previously validated method [13]. Animal procedures were approved by the Animal Ethics Sub-Committee of the University of KwaZulu-Natal (reference numbers 068/13/Animal and 025/14/Animal) [13]. A population pharmacokinetic model was constructed to describe the expected pharmacokinetic changes during repeated dosing. The serum and spleen drug concentration data were used as input into NONMEM (ver. 7.3.0) to determine the best predictive modeling approach based on the goodness of fit. To better understand the pharmacokinetics of CFZ prior to the phase transition, we created a collection of different (one-, two- and three-compartment), standard pharmacokinetics models, assuming drug stays in solution without precipitating in host organs. With these models, the data were fit during the first 4 weeks of CFZ dosing (Table S1), when a minimal amount of the drug was expected to precipitate as CLDIs. The model with the highest predictive accuracy in the soluble phase was then used to compare the steady-state predictions with the measured drug concentrations over the entire 20-week dosing interval.

### 2.2. Optimizing Compartmental Pharmacokinetics Modeling by Incorporating an Adaptive Vd Function

Four adaptive Vd models were used to capture different Vd expansion functions (linear, exponential Hill equation, logistic function, and rational square root sigmoid). The adequacy of each model was then tested and compared to the baseline, standard compartmental pharmacokinetics model with a constant Vd, to determine the best compartmental modeling approach and adaptive Vd function that fits the measured, temporal changes in the pharmacokinetics of CFZ over the 20-week treatment period. The baseline, standard compartmental model with a constant Vd is referred to as the “base model”. The predictive value of including a peripheral compartment to the base model was separately assessed to determine whether the phase transition can be captured by adding an additional compartment. Model superiority was determined by plotting the concentration predictions over time, comparing correlation of the residuals, coefficients of variation (CV%), and comparing Akaike information criterion (AIC) [15]. NONMEM models were constructed under the ADVAN9 subroutine using first-order conditional estimation with interactions (FOCEI), and a multiplicative error model was used across all evaluated models. Between-subject variability (ETA) was added to the growth function parameters involved in rate of expansion and maximum total expansion of the spleen volume.

### 2.3. Model Validation

**Figure 1:**
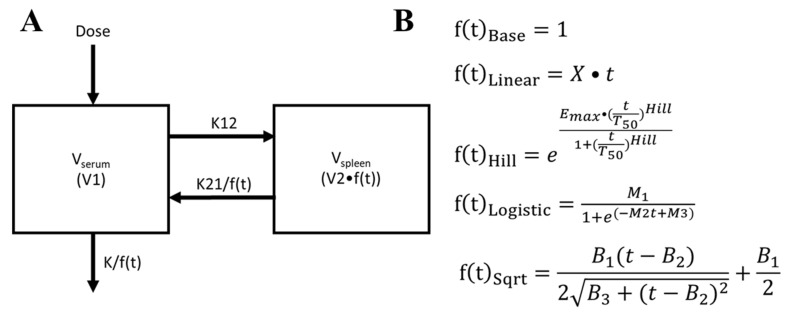
(**A**) The 2-comparment model used in this study. V_serum_ = V1, which represents the volume in the serum compartment, and V_spleen_ = V2•f(t) which represents the volume in compartment 2 multiplied by the expansion function f(t), respectively; K12 and K21 represent the intercompartmental rate constants; K represents the elimination rate constant; and f(t) represents the adaptive volume of distribution with respect to continued dosing. The f(t) function was modified to illustrate the effect of differing sigmoidal equations on the goodness of fit of the experimental data. (**B**) The five f(t) equations used to represent this compartmental model, where X = rate of f(t) expansion, Emax = maximum allowable expansion by f(t), Hill = speed of expansion over time, T50 = time at which 50% of the maximum is reached, M1 = maximum allowable expansion by f(t), M2 = steepness at inflection, M3 = lateral shift of the expansion curve, B1 = maximum allowable expansion by f(t), B2 = exact time of the inflection point, B3 = speed of expansion over time.

In pursuit of a robust estimation of the expansion of volume of distribution, a varying number of parameter estimates from the soluble phase model were used to fix the initial conditions of the full model with the expansion function. We will refer to the “soluble model” or “soluble phase model” as the two-compartment model without an expansion function, intended to capture the kinetics of clofazimine molecules in solution, prior to the phase transition leading to the formation of CLDI precipitates that result in drug accumulation in the spleen. Bootstrapping was then conducted on a subset of the models using 1000 runs in Wings for NONMEM (WFN). Parameter estimates, confidence intervals, standard deviations, and coefficients of variation were evaluated alongside histogram plots to determine the optimal expansion model (Figure S1). The chosen model with fixed V1 and K12 was then evaluated across each of the expansion functions in Figure 1, utilizing the entire 20-week dataset. Despite using the same structural compartmental model, the soluble phase model was evaluated alongside the base model. As the soluble model only extrapolates the concentration predictions from the first four weeks of data, the resulting predictions can be compared to the base model which evaluates CFZ pharmacokinetics over the course of the phase transition.

### 2.4. Variable Clofazimine (CFZ) Mass Sequestration

At any given timepoint, the cumulative dose fraction of CFZ sequestered in the spleen was estimated by multiplying the spleen weight by the predicted spleen concentrations of the composite sigmoidal models, to give the resulting mass of CFZ sequestered. This mass was then divided by the total dose of CFZ administered up until that timepoint, Equation (S2). The individual dose fraction sequestered is estimated by multiplying the spleen weight by the predicted concentration at each individual timepoint and subtracting the cumulative mass up until the previous timepoint. The mass sequestered between timepoints is then divided by the mass of the dose administered between the corresponding timepoints to estimate the fraction of dose sequestered Equation (S3). Mass balance was verified for each of the constructed models based on the predicted concentration values and measured organ weight.

### 2.5. Statistical Analysis of Experimental Data

The Akaike information criterion (AIC) reported in this article was calculated by multiplying the number of parameters by two and adding the reported objective function value (OFV) in NONMEM Equation (S1), and was one criterion used to determine model superiority. Statistical improvement in AIC was based on direct comparison between models. The correlation coefficient of predicted and observed concentration values was evaluated for incremental improvement over the base model. Means and standard deviations were calculated between pharmacokinetic parameters of sigmoidal functions to arrive at a consensus value. Bootstrapping analysis was conducted in WFN with 1000 runs per model. Parameter estimates, AIC and coefficients of variation were reported from the bootstrapping analysis. Statistics and regression completed outside of NONMEM were calculated in R Studio (Version 1.1.456).

## 3. Results

### 3.1. A Baseline, Compartmental Pharmacokinetics Model to Capture the Soluble-to-Insoluble Phase Transition of CFZ

From 11 different models, we selected the simplest compartmental model that best captured CFZ accumulation in the spleen: a bidirectional two-compartment model with elimination from the serum compartment. This model had the lowest objective function value and most physiological relevance of the 11 total compartmental models tested in the first four weeks of treatment (Table S2). The concentration data from both the serum and spleen were sampled into separate compartments in the model. Serum concentration data was sampled into the compartment which received the drug upon administration, and spleen concentration data were sampled into a second organ compartment that received drug from the blood. We only considered drug accumulation in the spleen, as most of the drug in the organism was found to accumulate in said organ over the 20-week treatment period. This two-compartment model was then modified to better capture the spleen and serum drug concentrations, over the full 20-week dosing period. To account for the non-linearity expected from CLDI formation, we added an adaptive function (V1) to the volume of the spleen compartment, the intercompartmental rate constant from compartment two to compartment one (K21), and the elimination rate constant (K). The compartmental model and associated adaptive Vd equations are shown in Figure 1. The predictions for the two-compartment model were directly compared to a three-compartment model with a serum, spleen, and peripheral compartment (Figure S2).

### 3.2. Optimizing the Two-Compartment Model by including an Adaptive Vd Expansion Function

The pharmacokinetic parameters of the base model and each of the four adaptive Vd modeling approaches that were used to refine the two-compartment modeling fits are shown in Table 1, alongside the AIC and R^2^ values. The base model behaved as a standard two-compartment model with a constant Vd. The linear model increased Vd linearly with respect to time, and the three sigmoidal models (Hill equation, logistic function, and rational square root sigmoid) all nonlinearly increased Vd with respect to time. The three-compartment model is identical to the base model with an additional peripheral compartment connected to the serum (Figure S2).

The base model had the fewest number of pharmacokinetic parameters and is therefore the simplest of the models tested in this study. The linear function had one additional parameter compared to the base model, and the three sigmoidal models, as well as the three-compartment model, all had three additional parameters compared to the base model. Due to the difference in number of parameters, significant improvement between models was determined by comparing AIC values rather than OFV.

**Figure 2:**
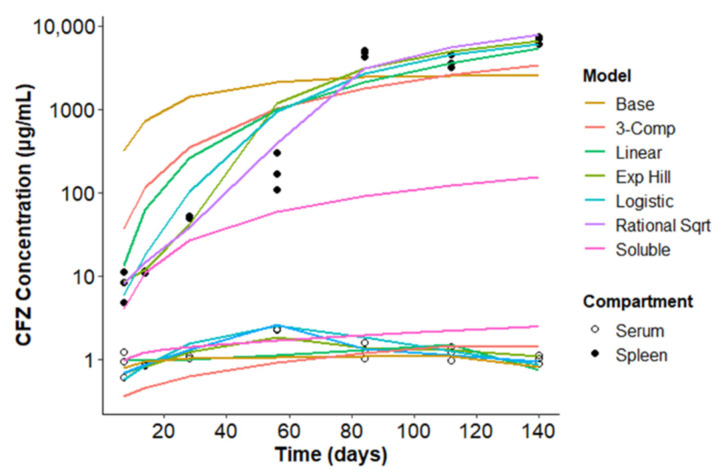
Visual predictive accuracy of CFZ concentration vs. time of four different expansion Vd models, the standard base two-compartment model, the three-compartment model, and the soluble phase model using the base model with data from the first 28 days of dosing with extrapolated predictions out to 140 days. The represented data were acquired from three mice for each serum and spleen timepoint, except at 14 days, where only two data points were available. The lines represent the corresponding concentration vs. time predictions. Experimental data obtained from three mice at each time interval are displayed on the graph alongside the average model predictions for the population.

The base model showed the lowest correlation coefficient and highest AIC, meaning of the five models, this one is statistically the least likely to predict the observed data. The linear model showed incremental improvement over the base model with a statistically significant decrease in AIC and increase in R^2^ (0.95 compared to 0.84). Incorporation of a sigmoidal function to capture the change in Vd over the treatment period improved all three models over the linear model (logistic function to rational square root sigmoid, respectively, Table 1). This finding indicates that any of the three sigmoidal equations could be used to better predict the soluble-to-insoluble phase transition compared to the base model or the linear model. The three-compartment model, used to fit serum and spleen concentration data, yielded a resultant AIC of 300.8 and R^2^ of 0.92, showing superiority over the base two-compartment model, but inferiority to all adaptive expansion models. The rational square root function showed the lowest AIC and had the highest R^2^ value of 0.99, reflecting the best fit to the data as observed by visual inspection (Figure 2). The Hill function almost fit the data as well as the rational square root function, with R^2^ of 0.98 and an AIC of 211.9. However, the CV% were smaller for each of the parameter estimates in the Hill function compared to the rational square root function. These two models were superior to all other models evaluated in this study. Most importantly, the pharmacokinetic parameter estimates converged to similar values when comparing the Hill and rational square root equation (Table 1), which were the two models that showed the best fit to the data.

Next, the accuracy of each of these four adaptive Vd models was evaluated by visual inspection of concentration versus time plots and direct comparison of the performance of the base two-compartmental model and the three-compartment model (Figure 2). During the 20-week time course of the experiment, CFZ concentration in the serum remained constant (~1 µg/mL) over the 20-week dosing period, while the CFZ concentration in the spleen increases in a nonlinear fashion over the duration of the experiment.

All six models utilizing the entire dataset accurately matched the observed serum concentrations. The base model overpredicted early and underpredicted late CFZ concentrations in the spleen. This trend indicates that the two-compartment base model inadequately captures the accumulation of CFZ in the spleen over time. The linear expansion function, which allows the Vd to expand linearly over time, yielded a more accurate fit than the base model (Figure 2). While still overpredicting the early time points and underpredicting the later timepoints, the linear Vd expansion equation improved upon the base model predictions, such that the simplest adaptive Vd function increased its predictive accuracy. At a higher level of complexity, using an exponential Hill equation, a logistic expansion function, or a rational square root equation as the adaptive Vd function, led to further improvement on the fits of the data. All three sigmoidal expansion functions more accurately captured the earlier and later timepoints than the linear or base models. Once again, the three-compartment model showed superiority in visual predictive accuracy to the base model, and inferiority to the adaptive Vd models.

The visual comparison of the extrapolated predictions of the base model using the first 28 days (soluble model) and the base model with the full dataset further reveal the inability of the base model to fully capture the range of concentrations over the course of dosing. The soluble model accurately captures the early spleen concentration values, but when extrapolated to the full course of dosing, vastly underpredicts the later spleen concentration values. The base model with the full dataset more closely captures the higher spleen concentrations at the later timepoints, but compensates by overpredicting the earlier spleen concentrations.

**Figure 3:**
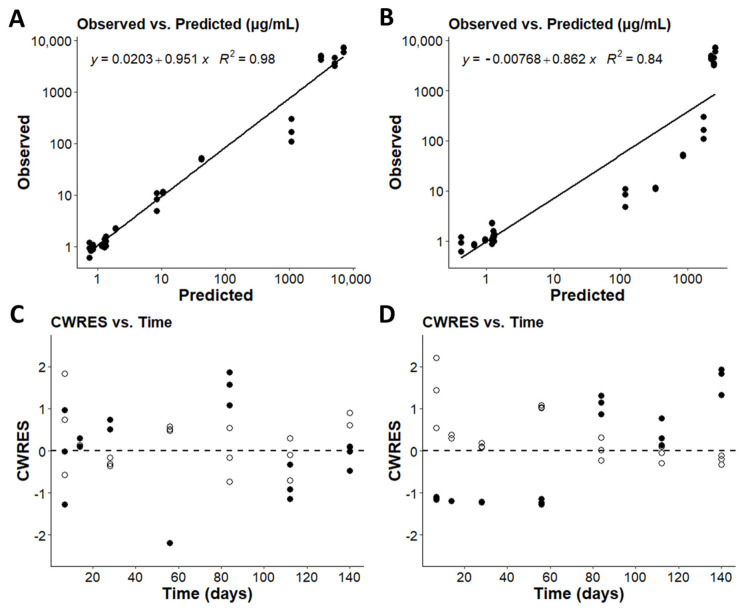
Observations vs. predictions (**A**,**B**) and the conditional weighted residuals vs. time (CWRES vs. Time; (**C**,**D**)) for the two-compartment model with the exponential Hill expansion function and the base two-compartment model. The weighted residuals for serum (open) and the weighted residuals for the spleen (closed) are plotted side by side. (**A**,**C**) are the diagnostic plots for the exponential Hill model. (**B**,**D**) are the diagnostic plots for the base two-compartment model.

We further analyzed the improved fits obtained with the models by incorporating a sigmoidal equation to capture the Vd expansion function. Quantitatively, the AIC of the Hill equation showed a significant quantitative improvement over the base model (Table 1). The Hill equation improved the observations versus predictions in the expansion model (Figure 3A) when compared to the base model (Figure 3B). The expansion model has an even distribution of residuals for both the serum and spleen concentrations without any clear trends. Whereas the base model shows underprediction of serum concentrations early in dosing, and an overprediction of spleen concentrations in the early timepoints with underprediction in the later timepoints. This improvement was also apparent in the weighted residuals from the Hill equation expansion model (Figure 3C) and the base model (Figure 3D). The quantitative improvements for each of the other Vd expansion functions and 3-compartmental model are also reflected in the residual plots (Figure S3).

Additionally, when comparing the fully optimized, unfixed models, the base model has a constant volume of distribution at 368.8 L/kg and a terminal half-life of 21 days, which is vastly different to the predicted volume and terminal half-life of the base model in the soluble phase (43.45 L/kg and 537.2 days, respectively). The exponentiated Hill function bridges the base model and soluble model pharmacokinetic parameters by allowing the soluble and insoluble phases to be incorporated simultaneously. The Vd of the Hill function increases from 62.7 L/kg to 147.9 L/kg, and half-life increases from 1.26 days to 343.0 days. This implies that the base compartmental model is unable to capture the entire dataset and requires an element of nonlinearity to predict the distribution of CFZ more accurately.

### 3.3. A Consensus Pharmacokinetics Model of the Expanding Vd of CFZ

Using all three sigmoidal equations to represent the adaptive pharmacokinetics of CFZ, two physiologically relevant pharmacokinetic parameters (Vd and half-life) were calculated to enable a consensus model that captures the drug concentrations and mass in the serum and spleen over the entire time course of the 20-week dosing regimen. For all models, the V_serum_ was kept constant, and the increase in the volume of the spleen was captured by each of the three sigmoidal equations. The base pharmacokinetic parameters (K, K12, K21, V1 and V2) for each of the models were fixed to the estimates from the fully optimized, unfixed exponential Hill function, allowing the adaptive parameters from the expansion function to distinguish amongst the sigmoidal models.

**Figure 4:**
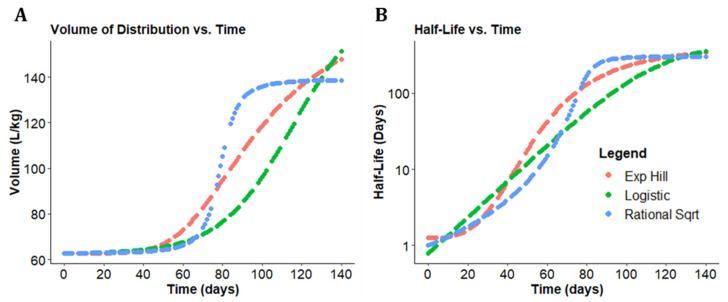
Changes in CFZ volume of distribution (Vd) and half-life over time. (**A**) The total Vd increased nonlinearly over the 20-week CFZ administration for each of the 3 sigmoidal functions. (**B**) The half-life over time increased for each of the sigmoidal equations over the 20 weeks of CFZ dosing.

The sum of the volume of the serum compartment and the spleen compartment was used to calculate a total Vd at each timepoint Equation (S4). The total Vd obtained for each of the models, incorporating the different sigmoidal functions, was then plotted over time, allowing for the inspection of Vd expansion variability with each sigmoidal model (Figure 4A). Each individual sigmoidal function was then used to plot the half-life over time (Figure 4B).

**Figure 5:**
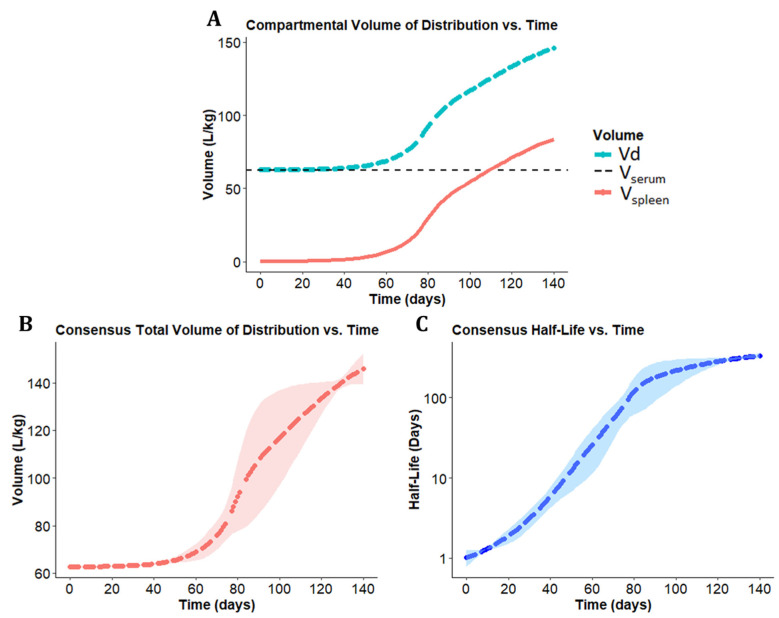
Changes in consensus values for CFZ total volume of distribution (Vd) and half-life over time. (**A**) The mean Vd is shown for each compartment. The dashed black line indicates the Vd contributed by the serum compartment, the red line indicates the Vd contributed by the spleen compartment, and the blue line indicates the total Vd of the drug. (**B**) Consensus total Vd over time with the shaded region indicating standard deviation. (**C**) The consensus half-life over time shown alongside the standard deviation.

To integrate the different models, the mean and standard deviation values of the pharmacokinetic parameters from the three sigmoidal functions were used to calculate composite values for total Vd and half-life (Figure 5B,C). The total Vd functions were also plotted alongside the Vd of the serum and spleen compartments (Figure 5A). Based on this consensus view, the serum compartment had a mean Vd of 62.5 L/kg, while the Vd of the spleen compartment increased from 0.26 L/kg to 86.48 L/kg. The resulting total Vd calculated by adding the serum and spleen compartment Vd increased 3-fold from 62.7 L/kg to 148.9 L/kg over the 20-week CFZ administration period. At each time point, the half-life was calculated based on first-order elimination Equation (S5). Accordingly, the mean consensus half-life increased from 1.05 days to 346.84 days.

These results indicate that there is an increasing amount of drug being sequestered over time, with an associated nonlinear increase in the Vd. The changes in half-life are modeled to be secondary to the changes in the Vd. The results shown here are driven by an adaptive volume in compartment two induced by a soluble-to-insoluble phase transition. Each sigmoidal expansion function predicts adaptive pharmacokinetic parameters as a surrogate for CLDI growth at varying rates and extents based on the supplied dataset. The overall range of values for total Vd at week 1 extended from 62.7 L/kg to 62.8 L/kg (for the Hill function and rational square root function, respectively), and a range for total Vd at week 20 from 143.9 L/kg to 155.1 L/kg (for the rational square root and logistic expansion function, respectively). The overall discrepancy in total Vd between the sigmoidal functions over the course of dosing spanned a 0.16% difference at initiation to a 7.5% difference at the end of the dosing, with the largest difference in functions (44.2%) apparent at 76 days. Similarly, the overall discrepancy in half-life between expansion functions ranged from a 42% difference at week 1 to a 12% difference at week 20, with the largest difference in functions (100.6%) apparent at 76 days.

As shown in Figure 4A, each sigmoidal curve optimizes to a different curve shape. The difference of fit comes from several sources. First, while each expansion function is a sigmoidal curve, each function differs slightly due to variation in curvature. That is, there is no way to set parameters of f(t)_Hill_ and f(t)_Sqrt_ such that f(t)_Hill_ = f(t)_Sqrt_ for all timepoints t. Additionally, the rational square root and logistic growth curves are symmetric about their inflection point, while the exponential Hill curve is asymmetric in that its inflection point occurs before the function reaches half capacity.

The maximum estimated expansion of the spleen compartment is a 347-fold increase based on the Emax parameter of the exponentiated Hill function, and the maximum rate of volume expansion occurred at 81.8 days based on the inflection point of the expansion function. The time at 50% maximum volume expansion was calculated to be 98.1 days after initiating dosing based on the plotted exponential Hill equation. At a 25 mg/kg dosing regimen, this corresponds to a total load of 35.0 mg, or 1.75 g/kg of total CFZ load, to reach 50% of maximum volume expansion. These predicted values alongside the estimated 273-fold increase in V_spleen_ at week 20 indicates that further increase in total volume is expected before reaching a loading capacity in the spleen. The resulting ETA values for the Hill coefficient and Emax parameters were very small (4.00 × 10^−6^ for both parameters) due to the homogenous mouse population and small sample size.

### 3.4. Analyzing CFZ Time-Dependent Pharmacokinetics and Mass Accumulation in the Spleen

**Figure 6:**
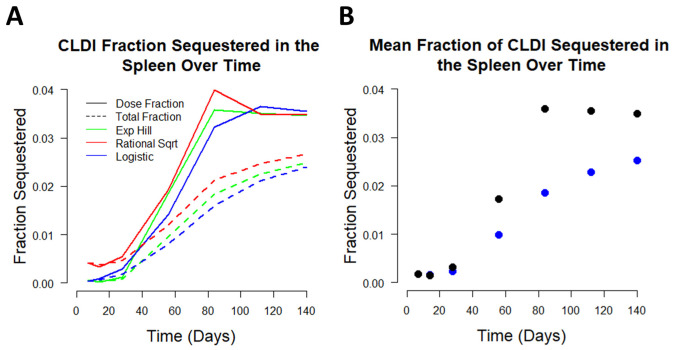
Sequestration of CFZ by the spleen. Dose fraction estimation was made by calculating the incremental CFZ mass increase with each dose compared to the predicted mass, to determine the change in CLDI sequestration over time. Estimation of cumulative fraction sequestered was made by calculating the fraction of administered CFZ present in the spleen. (**A**) Fraction of CLDI sequestered for both dose fraction and cumulative fraction for each of the 3 sigmoidal functions. (**B**) Cumulative fraction of all CFZ sequestered over time (blue) and estimated sequestered fraction of each individual dose given (black).

In addition to CFZ serum and spleen concentration predictions, we determined the fraction of the total dose of CFZ that bioaccumulates in the spleen over the 20-week treatment period. At each time point, the mass of CFZ calculated in the spleen by each of the sigmoidal expansion functions was divided by the cumulative dose of drug administered to mice up to that time point (Figure 6A). The consensus mass of CFZ in the spleen was calculated by averaging the predicted CFZ mass from each of the sigmoidal expansion functions (Figure 6B).

When given a CFZ dose of 25 mg/kg per day, the mean cumulative fraction sequestered in the spleen among the consensus sigmoidal functions increased from 0.0017 on week one to 0.025 on week 20, equating to a 14.7-fold increase in drug sequestration over the 20-week period. The estimated fraction of CFZ sequestered per dose increased from 0.0017 on week one to 0.035 on week 20 with a peak of 0.036 on week 12. The cumulative dose fraction between sigmoidal models was consistent with a 165% difference at week 1 and a 10.5% difference at week 20. The most pronounced change in the fraction of CFZ sequestered occurs between weeks four and eight, coinciding with the time during which CLDI formation becomes microscopically visible in mouse organs (at around 3 weeks of treatment), after which the spleen dramatically increases in mass (between 4 and 8 weeks of treatment) [9]. This observation is consistent with the increase in drug accumulating intracellularly within macrophages of the spleen as the insoluble drug precipitates. The time-course data show that an increasing fraction of administered drug is sequestered with each dose after 4 weeks of treatment. This is consistent with previous experimental observations indicating that CLDIs are visible in macrophages after 3 weeks of treatment, and then grow dramatically thereafter [9]. However, after 12 weeks of dosing, the estimated fraction of each dose that accumulates within the organism plateaus, suggesting that a maximum loading rate is reached at that time. This is consistent with the extent of CFZ accumulation within the host being determined by an active biological mechanism (e.g., the pH-dependent lysosomal acidification) that determines the upper limit of the rate at which the drug becomes sequestered within macrophage as the hydrochloride salt form.

## 4. Discussion

Our pharmacokinetic modeling of bioaccumulation shows the importance of considering phase transition kinetics in the evaluation of CFZ pharmacokinetic parameters. Of the six complete models evaluated in this study, the base two-compartment model performed the worst due to the inability to capture the insoluble precipitated phase. The three-compartment model improved upon predictions of the base two-compartment model as it could capture the additional distribution due to the CLDI phase as a static environment, but could not account for the variability of different amounts of precipitation at different total CFZ loads. The four expansion functions on the two-compartment model all outperformed the base two-compartment and three-compartment model since they were all able to adapt to the increasing sequestration of precipitated drug. The sigmoidal expansion functions performed comparably and superiorly to the linear growth model, implicating a nonlinear expansion of CFZ Vd during the initial 20 weeks of dosing.

The limited sample size and long sampling interval introduces some inherent limitations to this study. While mice were administered the drug orally, absorption was not considered in the model due to infrequent sampling early after dosing. The exponential Hill function and rational square root functions evaluated in this study yielded low AIC, excellent visual predictive accuracy, and physiologically relevant parameters consistent with the measured pharmacokinetics and the biological mechanisms of CFZ precipitation and bioaccumulation in mice. Despite the apparent improvements by the sigmoidal expansion functions, the ability to distinguish between minute differences in models was hindered by the size of the dataset, limiting the inspection of interindividual variability and complicating investigation of which sigmoidal function best models the behavior of CFZ. However, with more data, more detailed mechanistic models could be evaluated with other expansion functions. Additionally, only the pharmacokinetics of healthy mice we evaluated in this study. Further analysis is needed to explore the effects of infectious disease on the pharmacokinetics of CFZ. As macrophages are implicated in CFZ accumulation, an infectious agent may alter the context-dependent pharmacokinetics of the drug.

In mice, the accumulation and distribution of CFZ within the macrophages of different tissues is now a well-characterized phenomenon. CFZ undergoes phase transitions in vivo that are paralleled by changes in the pharmacokinetic parameters that govern the half-life and Vd of the drug. With increasing CFZ load, the fraction of drug sequestered as CLDIs increases, with an associated expansion in the Vd of the drug. This is a previously underappreciated phenomenon that demonstrates that, with prolonged dosing, a range of CFZ accumulation patterns over time could lead to very different drug efficacy and toxicity profiles over the course of dosing [8]. Based on the observed increase in CFZ concentration in the spleen and other organs, CFZ does not reach a steady state, even though serum concentrations are constant during the treatment regimen.

While this study presents an approach to modeling and analyzing the drug load-dependent pharmacokinetics profile of CFZ in mice, the parameter values obtained from mice may not directly correlate with parameter values in humans. Nevertheless, the phenomenon analyzed in this study bears relevance to the human situation, since it is known that CFZ precipitates and accumulates in macrophages of patients treated with CFZ [14,16,17]. Despite differences in clearance and blood flow between humans and mice, this modeling framework establishes an adaptive compartmental approach which requires no external, theoretical compartments, while also attempting to explain drug accumulation through the perspective of a context-dependent volume of distribution. From published articles showing CFZ crystals present in macrophages under a variety of total drug loads [18], we can safely assume that the principles of nonlinear, adaptive Vd expansion demonstrated in this study will apply to CFZ dosing regimens with associated phase transitions. The increasing Vd, driven by CFZ precipitation and bioaccumulation, indicates a need for re-evaluating methods by which steady-state drug concentrations in serum are interpreted and their relevance to the local drug concentrations at the site of action.

In relation to the three-compartment approach for modeling CFZ pharmacokinetics in humans, our study suggests that adding more peripheral compartments may indeed improve predictive accuracy, but not beyond that of adaptive Vd models. This indicates that as CFZ precipitates with continued dosing, dynamic modeling approaches more closely approximate CFZ pharmacokinetics, relative to fixed volume, multicompartment models. Although there is variability in the estimation of volume of distribution and half-life using the different sigmoidal equations, the different functions converge towards consensus parameter estimates that are all within a 2-fold range of values. This indicates that the structure of the sigmoidal function is less important than the model flexibility in adapting to the expanding volume of distribution.

CFZ loading capacity has been previously studied under a different dosing regimen [6]. The total CFZ load reported in this analysis was equal in mass to the cited study but administered over a 2.5-fold longer period, potentially leading to significantly different patterns of drug sequestration and clearance. Due to the difference in fractional sequestration of CFZ at different total CFZ loads, we would expect the pharmacokinetics and drug accumulation to change in a dose-dependent fashion. By combining classical compartmental kinetics with the mechanistic implications of soluble-to-insoluble phase transitions, CFZ accumulation rate and capacity can be estimated in an organ-specific accumulation pattern.

From these data, it is not clear whether CLDIs contribute to the pharmacological activity of the drug, or whether they may lead to toxicity, which may include changes in the immune system or metabolic changes that accompany CFZ accumulation [3]. Clearly, the increase in CFZ mass with repeated dosing corresponds to a drug depot mechanism that is poised to influence how long the drug remains in the host after treatment in discontinued. When selecting a dose for treatment, both the potential for therapeutic benefit and adverse events needs to be considered for optimal use of the drug. In the case of a fastidious mycobacterial infection, long half-lives can be important in terms of preventing drug-resistant microorganisms from taking a foothold, long after treatment is discontinued. Thus, this analysis serves to highlight many research avenues that still remain to be explored in the context of the pharmacokinetics and pharmacodynamics of CFZ, even as this drug has been used across the world for the elimination of leprosy for half a century (and now for the treatment of multidrug resistant tuberculosis and possibly for the treatment of viral infections).

This work may also constitute a wider modeling framework for bioaccumulating drugs or molecules with context-dependent pharmacokinetic properties. For example, azithromycin has been shown to accumulate intracellularly in human skin fibroblasts, implicating a cell-dependent accumulation pathway [19]. Likewise, amiodarone accumulates in alveolar macrophages, inducing phospholipidosis and potentially undergoing a context-dependent change in pharmacokinetics [20]. This unique approach to modeling allows for further study of drugs in the development pipeline which have poorly understood pharmacokinetic attributes. The generalization of this model to new and existing pharmaceutical compounds may improve the efficacy and toxicity profile of dosing regimens.

## 5. Conclusions

Using mice as an experimental model, and based on a dosing scheme of 25 mg/kg of CFZ daily, the changing pharmacokinetics of CFZ over a 20-week time course of treatment were modeled using an adaptive Vd function to capture the bioaccumulation of the drug in the organism. By incorporating nonlinearity into our pharmacokinetic models to capture soluble-to-insoluble phase transitions, it was possible to more accurately predict serum and tissue concentrations. It is likely that such adaptive pharmacokinetics modeling framework would also be applicable to the analysis of human clinical data, which could better inform our dosing strategies and understanding of CFZ efficacy and side effects. By analyzing the accumulation of CFZ within macrophages that can be obtained in patient sputum samples, it is possible that human pharmacokinetics models of CFZ could be improved. While drug accumulation in mice tissues is analyzed after the animals are euthanized, one avenue to explore, analyze, and model the extent of CFZ accumulation in human PK datasets could involve noninvasive pharmacoimaging techniques, such as positron emission tomography (PET).

## Figures and Tables

**Table 1:** Pharmacokinetic parameter estimates for each of the five 2-compartment models are represented alongside the R^2^ and AIC values for each corresponding model. The values for V2, K12 and K21 listed here are the initial values prior to the changes made by the f(t) equation. Variable 1 represents the slope X for the linear function, Emax for the exponential Hill function, M1 for the logistic function, and B1 for the rational square root function. Variable 2 represents the T50 for the Hill function, M2 for the logistic function, and B2 for the rational square root sigmoid. Variable 3 represents the Hill coefficient for the Hill equation, M3 for the logistic function, and B3 for the rational square root sigmoid. CV% are reported beside the parameter estimates for each model.

Model	R^2^	AIC	^ V1 (L/kg)	V2 (L/kg)	K (1/day)	^ K12 (1/day)	K21 (1/day)	Variable 1	Variable 2	Variable 3
Base Model	0.84	319.05	2.43	0.00516 (0%)	0.033 (0%)	0.183	0.00287 (33.8%)	N/A	N/A	N/A
Linear	0.95	255.8	2.43	0.0119 (32.8%)	0.275 (56.5%)	0.183	0.125 (49.1%)	* 0.219 (66.7%)	N/A	N/A
Exponential Hill Equation	0.98	211.9	2.43	0.00708 (1.61%)	0.229 (12.6%)	0.183	3.36 (1.53%)	* 6.99 (0%)	* 49 (2.68%)	3.71 (2.59%)
Basic Logistic Function	0.97	238.9	2.43	0.00779 (4.73%)	0.00645 (111.3%)	0.183	0.0762 (96.2%)	* 99.1 (56.2%)	* 0.0701 (2.82%)	8.85 (3.60%)
Rational Square Root Sigmoid	0.99	200.8	2.43	0.00533 (2.00%)	0.107 (54.1%)	0.183	2.16 (53.4%)	* 1150 (32.3%)	80.2 (3.20%)	* 8.84 (36.5%)

^ Fixed parameter estimates from soluble phase model. * Parameters with ETA values applied to the model.

## Data Availability

Not applicable.

## Supplementary Materials

The following are available online at https://www.mdpi.com/article/10.3390/pharmaceutics14010017/s1, Table S1: Soluble Phase Models, Table S2: Soluble Phase Model Results, Figure S1: Structural models considered for soluble model. Figure S2: 3-Compartmental Model and Associated Pharmacokinetic Parameters, Figure S3: Diagnostic Plots of Expansion Functions.
