# Supplementary material for: An Adaptive Biosystems Engineering Approach towards Modeling the Soluble-to-Insoluble Phase Transition of Clofazimine
Source: Pharmaceutics. 2021 Dec 22;14(1):17. doi: 10.3390/pharmaceutics14010017 (PMC8779763; doi:10.3390/pharmaceutics14010017)
Supplement: Supplementary file 1 [file pharmaceutics-14-00017-s001.zip › pharmaceutics-1442611-supplementary.pdf]

pharmaceutics

MDPI

Supplemental Materials: An Adaptive Biosystems Engineering Approach towards Modeling the Soluble-To-Insoluble Phase Transition of Clofazimine

Andrew R. Willmer, Steven Dunne, Rosemary Swanson, Deepak Almeida, Nicole C. Ammerman, Kathleen A. Stringer, Edmund V. Capparelli and Gus R. Rosania

Table S1. Soluble Phase Models .

Model	Model Description	Differential Equations
1a	1 - Compartment Serum Only	DADT(1) = - K*A(1))
1b	1 - Compartment Peripheral Only	DADT(1) = - K*A(1))
2a	2 - Compartment Unidirectional (C1 Elimination)	DADT(1) = - (K1*A(1) + K12*A(1))<br>DADT(2) = K12*A(1)
2b	2 - Compartment Unidirectional (C2 Elimination)	DADT(1) = - K1*A(1)<br>DADT(2) = K12*A(1) - K12*A(2)
3a	2 - Compartment Bidirectional (C1 Elimination)	DADT(1) = (K21*A(2)) - (K12*A(1) + K*A(1))<br>DADT(2) = K12*A(1) – (K21*A(2))
3b	2 - Compartment Bidirectional (C2 Elimination)	DADT(1) = (K21*A(2)) - (K12*A(1))<br>DADT(2) = K12*A(1) – (K21*A(2) + K*A(2))
4	2 - Compartment Bidirectional (C1 & C2 Elimination)	DADT(1) = (K21*A(2)) - (K12*A(1) + K*A(1))<br>DADT(2) = K12*A(1) – (K21*A(2) + K*A(2))
5a	2 - Compartment Split Administration (50:50)	DADT(1) = (K21*A(2)) - (K12*A(1) + K*A(1))<br>DADT(2) = K12*A(1) – (K21*A(2))
5b	2 - Compartment Split Administration (75:25)	DADT(1) = (K21*A(2)) - (K12*A(1) + K*A(1))<br>DADT(2) = K12*A(1) – (K21*A(2))
5c	2 - Compartment Split Administration (25:75)	DADT(1) = (K21*A(2)) - (K12*A(1) + K*A(1))<br>DADT(2) = K12*A(1) – (K21*A(2))
6	3 - Compartment (C1 Elimination)	DADT(1) = (K21*A(2)) - (K12*A(1) + K*A(1))<br>DADT(2) = (K12*A(1) + K32*A(3)) – (K21*A(2) + K23*A(2))<br>DADT(3) = (K23*A(2)) – (K32*A(3))

Compartmental Modeling of Soluble CFZ Pharmacokinetics Prior to the Formation of Precipitates

Prior to modeling the phase transition, soluble state CFZ concentration data (the first 4 weeks of dosing) was used to determine the pharmacokinetics of CFZ before significant CLDIs are seen *in vivo*. A soluble model was defined as a compartmental model without an expansion function, intended to model the kinetics of clofazimine prior to significant accumulation of CLDI precipitates. 11 compartmental models were tested to determine best fit for the soluble state. The corresponding names and model number are listed in the supplemental table 1. All models were constructed in NONMEM using a multiplicative error model in ADVAN9 with the associated differential equations for each compartment listed in table 1. Each soluble phase model was evaluated on the basis of objective function and visual predictive accuracy (supplemental table 2). Model 1a and 1b did not contain the entire dataset and were not considered for the full phase transition model. The remaining nine models were similar in fit and OVF values. Model 3a (2-compartment bidirectional model with elimination through compartment 1) was selected for the full phase transition model due to the lowest OVF value and physiologically relevant compartmental structure.

Table S2. Soluble Phase Model Results.

Model	OFV	K (day^{-1})	K12 (day^{-1})	K21 (day^{-1})	V1 (L)	V2 (L)	K2 (day^{-1})	K23 (day^{-1})	K32 (day^{-1})
1a	-67.46	0.211	--	--	2.67	--	--	--	--
1b	93.49	0.00129	--	--	--	0.504	--	--	--
2a	53.32	0.189	1.24	--	0.233	0.373	--	--	--
2b	53.52	0.00129	1.23	--	0.305	0.421	--	--	--
3a*	48.42	0.0948	1.23	0.0145	1.26	0.31	--	--	--
3b	49.92	0.00129	1.06	0.01	0.498	0.411	--	--	--
4	49.87	0.00129	1.03	0.0102	0.521	0.409	0.00129	--	--
5a	50.44	0.123	0.061	0.00109	2.02	0.266	--	--	--
5b	49.65	0.00129	0.813	0.0193	1.26	0.416	--	--	--
5c	49.95	0.00129	0.35	0.00401	0.729	0.44	--	--	--
6	54.44	0.664	4.4	0.00142	0.0613	1.09	--	0.667	11.3

Further soluble phase modeling was conducted on model 3a (Table S2) to supply the full phase transition model with relevant fixed estimates to reduce complexity and improve confidence in the selected model. Soluble models were tested with model 3a (Figure S1A) and a parameterized model (Figure S1B).

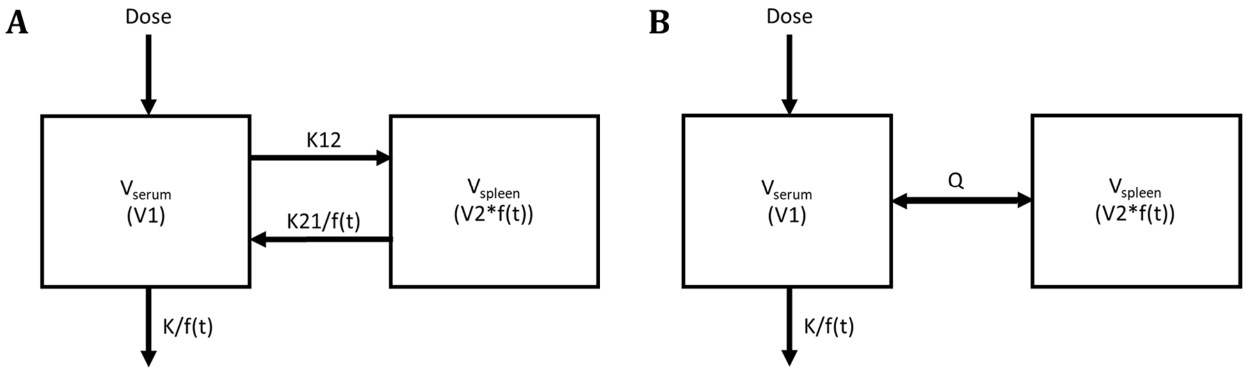

Figure S1. Structural models considered for soluble and phase transition model. A) Unparameterized bidirectional model B) Bidirectional model with parameterized intercompartmental rate constants. For the soluble models, f(t)=1, for the expansion models, f(t) is set to the exponential Hill function.

Four soluble models were constructed using previously published CFZ concentration data in healthy BALB/c mice in the first four weeks treatment [13]. Soluble model A used the unparameterized structural model (Figure S1A) with the first 4 weeks of data from the 20 week dosing study [13]. Soluble model B used the unparameterized model with the first 4 weeks of data and single dose concentrations in healthy mice [13]. Soluble model C used the parameterized structural model (Figure S1B) and the first 4 weeks of data. And finally soluble model D used the parameterized structural model alonside the first 4 weeks of data and single dose concentrations. The resultant parameter estimates from each of the soluble models were used as initial conditions for each of the full phase transition models. The number of fixed parameters were varied to determine the best subset of full models to run a bootstrapping analysis. Soluble model B was determined to be superior to soluble models A, C, and D from resultant AIC, and visual predictive accuracy. Bootstrapping analysis was then conducted on nine full phase transition models from the fixed estimates of the soluble model B using the exponential Hill function as the expansion function. From the resulting bootstrap analysis of the nine full phase transition models, the unparameterized model with fixed V1 and K12

performed the best based on 95% confidence intervals, coefficients of variation, and histogram plot distribution. This model was then used for comparative analysis with different expansion function equations (Table 1).

Incremental Compartment Modeling

By incrementally increasing the number of compartments, a peripheral compartment (V3) was added to the base model. The chosen 3-compartment model structure alongside the resulting objective function value, correlation of residuals, and PK parameters are shown in supplemental figure 2. The 3-compartment model was derived from an established pharmacokinetic model of CFZ. [12] The OFV, R^2, and distribution of residuals (supplemental figure 3) are correlated with an improvement upon the base model when supplied the entire data set, and inferior predictive capacity compared to the adaptive Vd models.

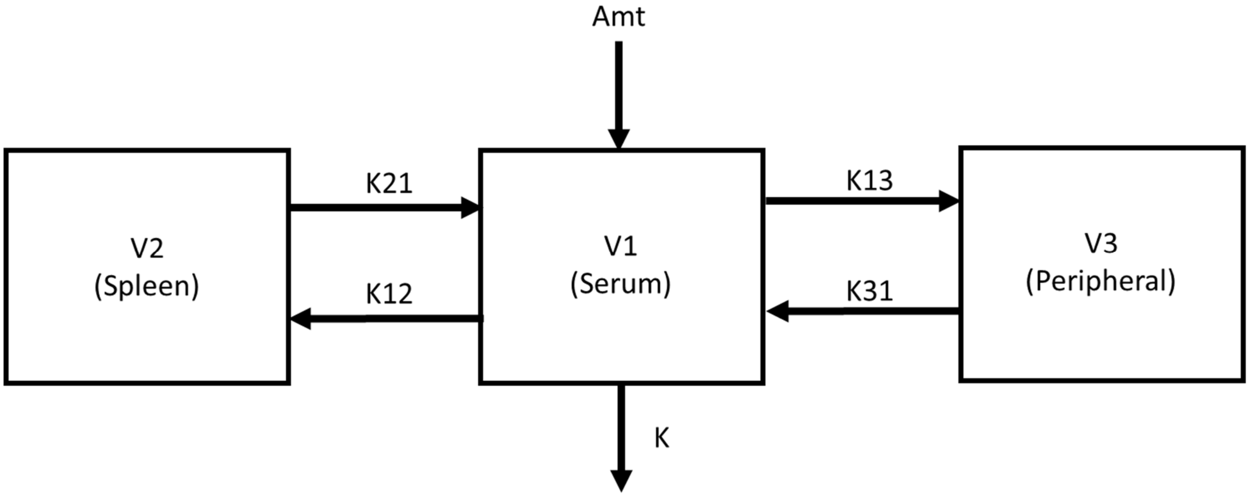

DADT(1) = (K21*A(2) + K31*A(3)) - (K12*A(1) + K13*A(1) + K*A(1))
DADT(2) = (K12*A(1)) - (K21*A(2))
DADT(3) = K13*A(1) - (K31*A(3))

AIC	R^2	K (day^{-1})	^K12 (day^{-1})	K21 (day^{-1})	K31 (day^{-1})	K13 (day^{-1})	^V1 (L)	V2 (L)	V3 (L)
300.8	0.92	0.007 (165.6%)	0.183	0.0225 (0%)	57.8 (164.6%)	603 (157.8%)	2.43	0.00622 (9.9%)	0.56 (0%)

^Fixed parameter estimates from soluble phase model

Figure S2. 3-compartmental model structure and associated pharmacokinetic parameters. The differential equations for the model and the predicted PK parameters are listed below the diagrammatic view of the 3-compartment model. The parameter estimates are provided alongside the respective coefficients of variation. The combination of AIC, CV% and R^2 indicates superiority to the base model, and inferiority to all four adaptive Vd models.

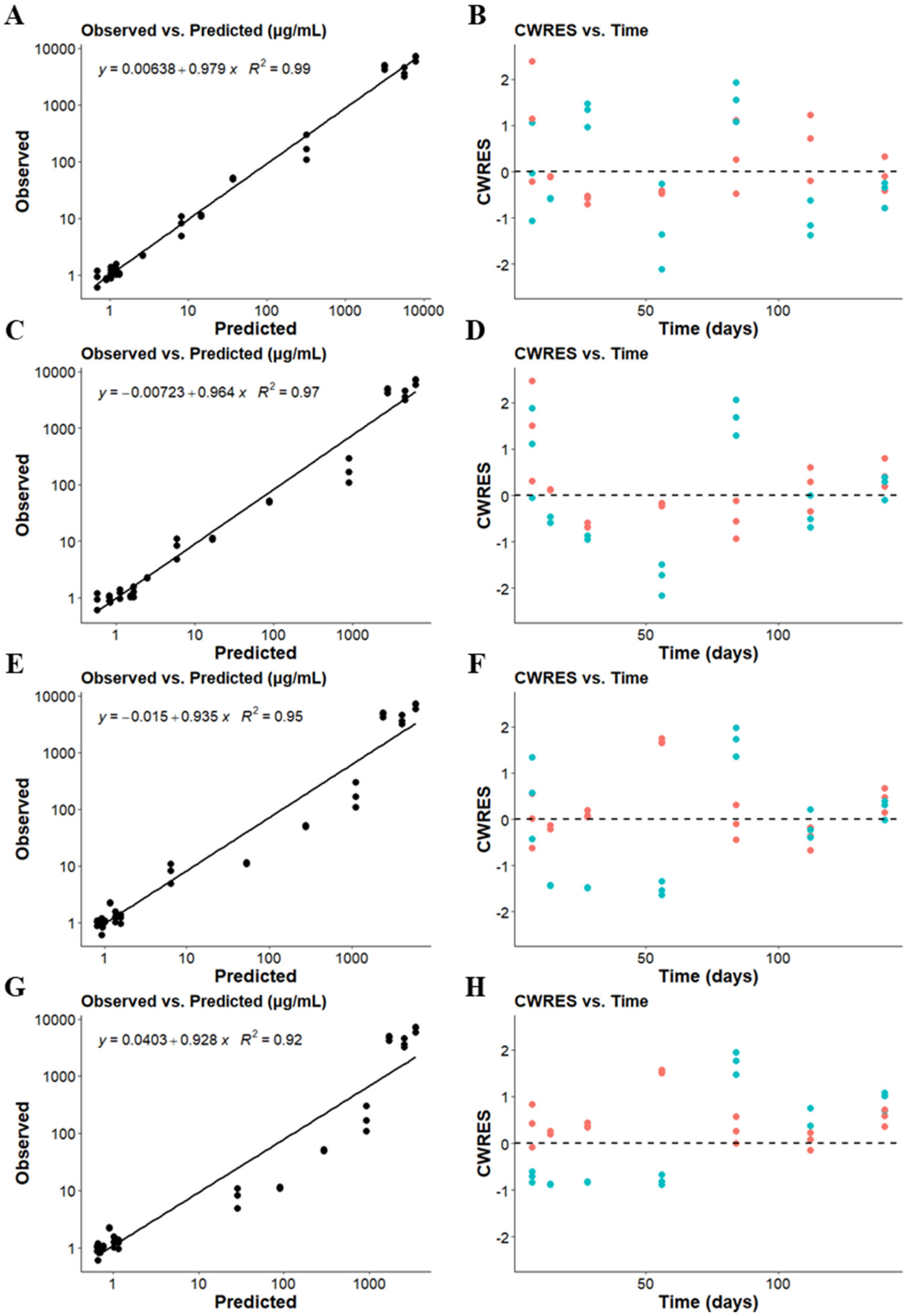

Figure S3. A and B are residual plots of the rational square root function. C and D are residual plots of the log growth function. E and F are plots for the linear function. And plots G and H are residual plots of the 3-compartment model. A, C, E, and G are plots of the observations vs. predictions and B, D, F, and H are plots of the conditional weighted residuals.

Supplemental Equations

$$AIC = 2 * K + OFV \qquad \text{(S1)}$$

The Akaike information criterion was calculated by multiplying 2 times the number of parameters (K) plus the resultant objective function value (OFV) reported in NONMEM [15].

$$DF_{comb}(t) = \frac{Mass_{spleen} * Conc_{pred}(t)/\delta}{Dose_{total}(\mathrm{t})} \qquad \text{(S2)}$$

Cumulative dose fraction ($DF_{comb}(t)$) was calculated to estimate quantity of dose expected to be sequestered as drug is continuously loaded into mice at each timepoint in weeks. Where $Mass_{spleen}$ is the mass of the spleen, $Conc_{pred}(t)$ is the predicted concentration at timepoint t, and $Dose_{total}(t)$ is the total administered dose up until timepoint t. The average mass of the spleen in the experiment is 0.179g and the density (δ) of the spleen was assumed to be 1.0 g/mL.

$$DF_{ind}(t) = \frac{Mass_{spleen} * Conc_{pred}(t)/\delta - Mass_{Drug}(t^*)}{Dose_{total}(t - t^*)} \qquad \text{(S3)}$$

Individual dose fraction ($DF_{ind}(t)$) was calculated to estimate the quantity of a single dose that is sequestered as the total sequestered mass is increased. The predicted dose fraction since the previous timepoint is divided by the total dose since the previous time point. Where t is the time in weeks, and t* is the time in weeks at which the previous measurement was recorded.

$$Vd = V1 + V2 + \cdots + Vn \qquad \text{(S4)}$$

Vd is the total volume of distribution calculated by the sum of the volume in all compartments. Most models used in this study were 2 compartment models.

$$T_{\frac{1}{2}} = 0.693/K_e \qquad \text{(S5)}$$

Half-life was determined based on 1st order elimination.

References

See attached manuscript for reference details.
